# Motor Cortical Plasticity in Parkinson’s Disease

**DOI:** 10.3389/fneur.2013.00128

**Published:** 2013-09-04

**Authors:** Kaviraja Udupa, Robert Chen

**Affiliations:** ^1^Division of Neurology, Department of Medicine, University of Toronto, Toronto, ON, Canada; ^2^Division of Brain, Imaging and Behavior – Systems Neuroscience, Toronto Western Research Institute, Toronto, ON, Canada

**Keywords:** M1 plasticity, Parkinson’s disease, transcranial magnetic stimulation, paired associative stimulation, theta burst stimulation, transcranial direct current stimulation, repetitive transcranial magnetic stimulation

## Abstract

In Parkinson’s disease (PD), there are alterations of the basal ganglia (BG) thalamocortical networks, primarily due to degeneration of nigrostriatal dopaminergic neurons. These changes in subcortical networks lead to plastic changes in primary motor cortex (M1), which mediates cortical motor output and is a potential target for treatment of PD. Studies investigating the motor cortical plasticity using non-invasive transcranial magnetic stimulation (TMS) have found altered plasticity in PD, but there are inconsistencies among these studies. This is likely because plasticity depends on many factors such as the extent of dopaminergic loss and disease severity, response to dopaminergic replacement therapies, development of l-DOPA-induced dyskinesias (LID), the plasticity protocol used, medication, and stimulation status in patients treated with deep brain stimulation (DBS). The influences of LID and DBS on BG and M1 plasticity have been explored in animal models and in PD patients. In addition, many other factors such age, genetic factors (e.g., brain derived neurotropic factor and other neurotransmitters or receptors polymorphism), emotional state, time of the day, physical fitness have been documented to play role in the extent of plasticity induced by TMS in human studies. In this review, we summarize the studies that investigated M1 plasticity in PD and demonstrate how these afore-mentioned factors affect motor cortical plasticity in PD. We conclude that it is important to consider the clinical, demographic, and technical factors that influence various plasticity protocols while developing these protocols as diagnostic or prognostic tools in PD. We also discuss how the modulation of cortical excitability and the plasticity with these non-invasive brain stimulation techniques facilitate the understanding of the pathophysiology of PD and help design potential therapeutic possibilities in this disorder.

## Plasticity – Long-Term Potentiation and Long-Term Depression

**Figure 1:**
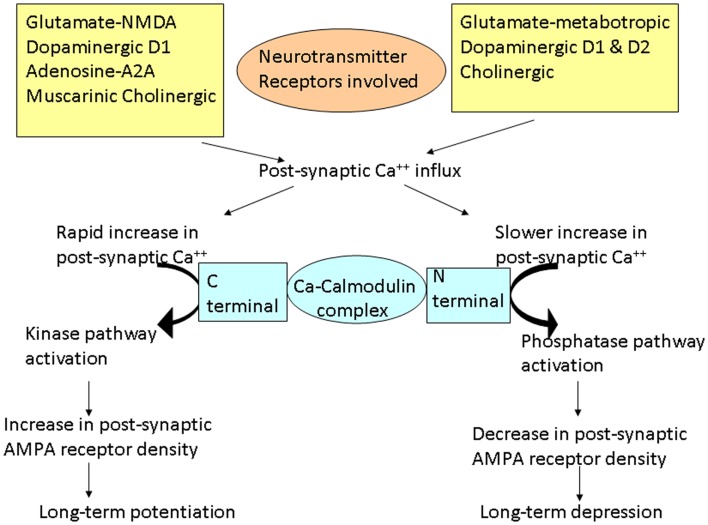
**Schematic representation of the cascades of events involved in long-term potentiation (LTP) and depression (LTD)**. Different neurotransmitters are involved in these cascades. Different changes occur depending on the rate of increase of post-synaptic calcium (Ca^++^). Rapid influx of Ca^++^ preferentially promotes binding of Ca^++^ to the C-terminal of calmodulin, activating the kinase pathways. These reactions lead to increase in AMPA receptor density on the post-synaptic membrane resulting in LTP. On the other hand, slower release of Ca^++^ leads to Ca^++^ binding to the N-terminal of calmodulin, activating the phosphatase pathways. This leads to decrease in AMPA receptor density on the post-synaptic membrane, resulting in LTD.

The word plasticity is derived from Spanish word “plasticina” meaning “play-doh” describing the property of a substance being impressionable or changes the structure or function depending on the situation. Neuronal plasticity refers to the ability of the neuron to modify its structure or functions in response to stimuli and these modifications outlast the stimulation period (1). These changes generally occur in the synaptic functions, thus modifying the interneuronal connections and is termed synaptic plasticity (2–4). These changes encompasses all possible mechanisms of neuronal network reorganization, including recruitment of pathways that are functionally homologous but anatomically distinct from the original ones, reinforcement of existing synaptic connections, dendritic arborization, and synaptogenesis (5). Such stimulation-induced modifications in synaptic efficacy, such as long-term potentiation (LTP) and long-term depression (LTD), represent key cellular substrates for adaptive motor control and procedural memory as demonstrated in animal models (6). LTP is generally defined as long-lasting but not necessarily irreversible increase in synaptic strength and LTD refers to decrease in synaptic strength (7). Induction of LTP and LTD depends on *N*-methyl d-aspartate (NMDA) receptor activation by glutamate and post-synaptic calcium influx (8). A rapid increase in post-synaptic calcium concentration binds the C-terminal of calmodulin and triggers a kinase pathway that increases the density and conductance surface α-amino-3-hydroxy-5-methyl-4-isoxazole propionic acid (AMPA) receptors leading to LTP (Figure 1). In contrast, a slower increase in calcium concentration promotes binding to the N-terminal of calmodulin, which operates via the phosphatase pathway and has opposite effect on surface AMPA receptors leading to LTD (9).

## Parkinson’s Disease

Parkinson’s disease (PD) is a progressive neurodegenerative disorder with degeneration of nigrostriatal dopaminergic neurons in basal ganglia (BG) resulting in a movement disorder characterized by tremor, rigidity, bradykinesia, and postural instability (10). According to the Braak model of PD (11), there is degeneration of other areas such as the brainstem in the early stages of the disease and in widespread regions including the neocortex in late stages and these changes lead to non-motor features of PD. Previous studies have demonstrated altered plasticity in BG related subcortical structures and in the primary motor cortex (M1) in animal and human studies in various stages of PD.

**Figure 2:**
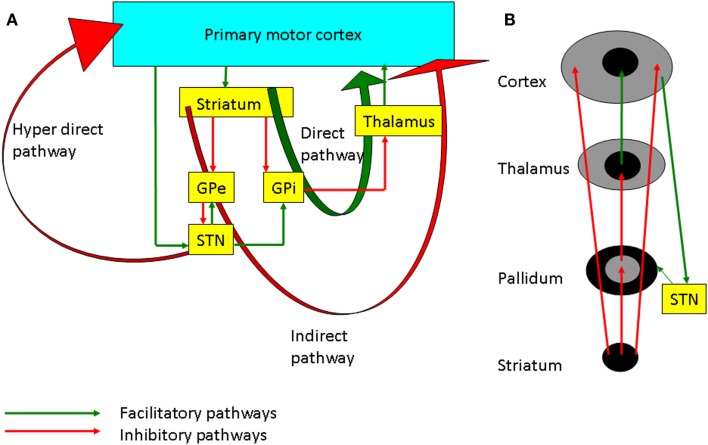
**(A)** The basal ganglia-thalamocortical loops involved in motor control. The internal globus pallidus (GPi) is the main output nucleus of the basal ganglia and it has inhibitory projection to the thalamus. The direct pathway projects from the striatum to the GPi. Inhibition of the GPi facilitates movement by increasing thalamocortical projections. On the other hand, the indirect pathway through the external globus pallidus (GPe), subthalamic nucleus (STN), GPi, and thalamus inhibits the excitatory thalamocortical output. The hyperdirect pathway through cortico-subthalamic nucleus projection is considered to suppress motor programs through facilitation of the GPi. **(B)** Schematic diagram showing the center facilitation surround inhibition model. The direct pathway shown in the center facilitates the movement whereas the indirect pathway in periphery of the projection inhibits the competing motor patterns for the specific movement. STN modulates the cortex through both the hyperdirect and the indirect pathways.

There are three main pathways of information processing in the cortico-BG loop (Figure 2A), the direct (cortico-striato-pallidal/nigral) and indirect (cortico-striato-pallido-subthalamo-pallidal/nigral) pathways via the striatum and the hyperdirect (cortico-subthalamo-pallidal/nigral) pathway via the subthalamic nucleus (STN) (12). *In vivo*, cortical activation leads to a triphasic synaptic response in SNr neurons, due to the sequential involvement of the three pathways: a first excitation, due to hyperdirect pathway activation, followed by an inhibition due to activation of the direct pathway and a late excitation from the indirect pathway (13). Functionally, it has been hypothesized that the hyperdirect pathway suppresses motor programs, allowing the direct pathway to select the goal-directed behavior, while the indirect pathway completes the motor activity (14). There are other models such as the center surround model (Figure 2B), which hypothesize that the direct pathway is the excitatory center, and the indirect pathway is related to the inhibitory surround (15). The management strategies of PD involve the use of dopaminergic medications and deep brain stimulation (DBS) which alter plasticity in both BG and M1, and these will be discussed in the following sections.

## Animal Models of PD and Studies of Synaptic Plasticity

A commonly used rodent model in PD is prepared by injection of 6-hydroxy dopamine (6-OHDA) into the striatum. This causes degeneration of dopaminergic neurons, which leads to alterations in striatal synaptic neurotransmission and plasticity. This dopaminergic deficit leads to increased glutamatergic activity and Ca^2+^-induced degeneration of dendritic spines of medium spiny neurons (MSN), which is an important site of the synaptic plasticity (16). MSN constitute 95% of striatal neurons and receive inputs from glutamatergic corticostriatal neurons and dopaminergic nigrostriatal neurons in addition to cholinergic interneurons. These synapses on dendritic spine of MSN undergo both LTP and LTD in both *in vitro* and *in vivo* (17) experiment models. Pharmacological studies showed that NMDA, dopaminergic D1 (mainly direct pathway), adenosine A2A (mainly indirect pathway), and muscarinic cholinergic receptors are involved in the induction of LTP (18, 19). The molecular process involves D1-mediated NMDA receptor (NR) complex modification through different neurotransmitter systems and finally inserting new AMPA receptors post-synaptically (Figure 1). On the other hand, activation of D1 and D2, metabotropic AMPA glutamatergic and cholinergic receptors may be required for LTD (19, 20). Endogenous release of dopamine has a role in determining the plasticity direction such that phasic release of dopamine favors LTP where as tonic release induces LTD (21). Dopamine deficit causes an over activity of the glutamatergic cortical to MSN projection expressed in part as an increase in spontaneous activities of MSN. Thus, dopamine depletion in PD alters the induction of LTP at these glutamatergic synapses (18).

Spike-timing-dependent plasticity (STDP) is another concept to explain bidirectional modulation of synaptic plasticity (4, 22). According to this theory, the generation of LTP or LTD depends on the timing between the activation of pre and post-synaptic cells. Classical Hebbian STDP involves the generation of LTP when presynaptic spikes precede post-synaptic spikes by up to 20 ms while LTD is induced when post-synaptic spikes lead presynaptic spikes by up to 100 ms (23). On the other hand, in anti-Hebbian STDP, pre-leading-post spike order drives LTD and post-leading-pre spiking drives LTP. This form of STDP opposite to Hebbian STDP was observed in MSN in BG (24) and in the somatosensory cortex (25).

It has been demonstrated that an imbalance between dopamine D2 and NR activities induce altered synaptic plasticity at corticostriatal synapses (26). α-calcium-calmodulin-dependent protein kinase II (α-CaMKII) functions as signal integrator (27) for these two neurotransmitters (glutamate and dopamine) related pathways and increased autophosphorylation of this molecule was associated with defective synaptic plasticity which parallels the development of motor abnormalities in Parkinsonian rats (28). This alteration of plasticity involved the absence of both LTP (29, 30) and LTD (31) in striatum and was postulated as the molecular mechanisms responsible for motor and cognitive symptoms of PD (6). In animal models of PD, complete dopaminergic denervation decreases both LTP and LTD but incomplete dopaminergic loss leads to decreased LTP in corticostriatal neurons (32) while chronic l-DOPA treatment restored LTD but not LTP types of synaptic plasticity (29).

## Measurement of Motor Cortical Plasticity Using Transcranial Magnetic Stimulation

Non-invasive brain stimulation technique such as transcranial magnetic stimulation (TMS) has been used to quantify various neurophysiologic measures in neurological and psychiatric disorders and has the potential to be used as a diagnostic and prognostic tool (33). Various TMS protocols have been used to induce LTP- and LTD-like changes in the brain and they may have therapeutic utilities in movement disorders (34). Depending on the direction of change in excitability, these protocols have been broadly divided into LTP-like and LTD-like protocols, which increase or decrease the excitability. Protocols such as intermittent theta burst stimulation (iTBS), high-frequency rTMS, and paired associative stimulation at 25 ms (N20 latency + ∼3–5 ms; PAS25) are considered LTP-like protocols. On the other hand, continuous (c) TBS, low-frequency rTMS, and PAS10 (PAS with 10 ms between peripheral nerve stimulation and M1-TMS) are considered LTD-like protocols as they decrease the excitability of the motor cortex. However, the mechanisms by which these protocols induce the specific type of plasticity (LTP or LTD) are different. Although PAS25, high-frequency rTMS, and iTBS induced LTP-like changes in M1, the process of induction of plasticity, time courses and the mechanisms involved are different (35). In general, plasticity induced by protocols that activate multiple sets of synapses (such as PAS acting through sensory-motor communications and intracortical circuits of M1) is termed as heterosynaptic plasticity. This type of plasticity depends on spike-timing-dependent mechanisms of activating pre and post-synaptic terminals within a time window as discussed earlier. This is different from homosynaptic plasticity (e.g., rTMS and TBS), which is induced by stimulating the same set of synaptic connections repeatedly and the effects are related to the frequency of stimulation (36). Though the molecular mechanisms of these non-invasive brain stimulation protocols involving homo and heterosynaptic plasticity have not been elucidated, we could infer their mechanisms based on similar protocols in slice preparations in animal models. Furthermore, the effect of sensory inputs are altered in PD (37, 38). Since PAS involves sensory input and rTMS does not, M1 plasticity probed by PAS and rTMS protocols may have different results.

Transcranial direct current stimulation (tDCS) has also been used to induce M1 plasticity. Anodal cortical tDCS typically induces LTP-like changes while cathodal tDCS induces LTD-like effects (39). Although the changes in the membrane excitability have been proposed as potential mechanisms of these changes, the molecular basis of this plasticity has not been fully determined. Thus, protocols employing tDCS to investigate M1 plasticity may involve mechanisms different from those using PAS and rTMS.

When two plasticity protocols are used one after another, the effect of first one modulates that of the second and this interaction has been termed as metaplasticity. Homeostatic plasticity is a concept to explain this interaction and is based on the principle that physiological systems attempt to maintain homeostasis to prevent excessive unidirectional changes. Therefore, when two LTP-like protocols applied consecutively, instead of further potentiating the effects, second protocol may bring the excitability back to the baseline. A similar concept is depotentiation. It refers to a protocol which on its own does not induce changes in the excitability but cancels the effect of a preceding potentiation protocol in order to achieve the homeostasis, which is maintained by the ratio of NR subtypes (NR1 and NR2) (40, 41). Thus, the comparison of different M1 plasticity studies employing different plasticity induction protocols or combination of protocols should take these differences into account.

## Variability of TMS Measures

Transcranial magnetic stimulation measures are variable and this has been widely reported (42–49). In addition to the well known pulse-to-pulse variation in TMS response which is partly due to spontaneous variation in cortical excitability, there are various intrinsic (genetic polymorphisms of neurotransmitters and receptors, hormonal level, attention level, fatigue of subjects) and extrinsic (coil placement, coil and stimulator parameters) factors responsible for this variation (50). These factors should be considered in the interpretation of plasticity studies in PD. Furthermore, the parameters used to measure plasticity are different in different studies. Many studies (Table 1) used stimulation intensity to generate 1 mV motor evoked potential (MEP) amplitude in intrinsic hand muscles before the plasticity protocol and used the same intensity to assess changes in cortical excitability where other studies used motor threshold, recruitment curve (input-output curve, MEP amplitudes with increasing stimulation intensities), 120% resting motor threshold, intracortical circuits, silent period, or behavioral measures. Since each parameter has its own strengths and drawbacks, one has to be vigilant when pooling the results of studies that used different parameters.

**Table 1:** **Studies measuring M1 plasticity in PD with TMS plasticity protocols**.

Study	Protocol(s) used	*n*	Age	Dis dur (y)	UPDRS-III (OFF)	H and Y	l-DOPA	LID	Main findings
**PAS**									
Morgante et al. (51)	PAS21.5	16	70 ± 5	9 ± 3	26 ± 10	2.3 ± 0.5	ON and OFF	7−	Decreased LTP in patients with OFF condition, improved with medication in patients without dyskinesias but not with LID
			67 ± 9	12 ± 5	29 ± 7	2.9 ± 0.8		9+	
Ueki et al. (52)	PAS25	18	65 ± 9	5 ± 3	19 ± 8	2–3	ON and OFF	−	Dopaminergic medications restored the impaired plasticity in PD although not to the level of healthy subjects
Bagnato et al. (53)	PAS25	16	63 ± 9	8 ± 4	–	2–3	ON and OFF	±	Exaggerated and overflow of M1 plasticity during OFF, normalized by medications; heterogeneous sample, more and less affected side not identified
Schwingenschuh et al. (54)	PAS25	25	69 ± 8	7 ± 3	28 ± 12	–	OFF	−	Deficient plasticity in PD which is different from healthy subjects (normal plasticity) and dystonia, scans without dopaminergic deficit and essential tremor. All three patient groups had exaggerated plasticity
Kojovic et al. (55)	PAS25	16	59 ± 3	2 ± 0.3	15 ± 2	–	–	DN	Impairment of plasticity on the more affected side, exaggeration of plasticity on the less affected side
Kacar et al. (56)	PAS25	20	52 ± 12	3 ± 2	32 ± 11	2 ± 1	OFF	10	M1 plasticity is equally deficient in drug-naïve and patients taking dopaminergic drugs
			55 ± 13	5 ± 4	31 ± 12	2.4 ± 1		DN 10−	
Kishore et al. (57)	PAS25	16 + 20	55 ± 2	9 ± 1	40 ± 4		ON and OFF	+	Deficient PAS-induced plasticity in PD patients with LID is restored by inhibitory cTBS to the cerebellum
			55 ± 2	11 ± 1	42 ± 5	
**TBS**									
Eggers et al. (58)	cTBS	8	69 ± 5	4 ± 3	26 ± 7	2 ± 1	OFF	−	No decrease in cortical excitability after cTBS
Benninger et al. (59)	iTBS (sham)	26	62 ± 7	11 ± 7	–	3 ± 0.4		−	Increase in MEP after first session of iTBS. No change in clinical parameters except mood improvement
Suppa et al. (60)	iTBS	20	62 ± 8	5 ± 4	26 ± 9	2.5	ON and OFF	11−	Decreased potentiation with iTBS and no difference with medication and LID
			63 ± 7	9 ± 5	29 ± 9	3		9+	
Stephani et al. (61)	iTBS	8	62 ± 8	–	–	1–2	ON	−	No changes in excitability with iTBS
Zamir et al. (62)	iTBS	12	65 ± 10	7 ± 3	23 ± 9	–	ON and OFF	7− 5+	Normal response in PD. ON medication showed increased in cortical excitability within 20 min after iTBS compared to OFF medication condition
Kishore et al. (63)	iTBS cTBS	10	51 ± 4	3 ± 1	12 ± 1	–	ON and OFF	−	Both protocols did not elicit changes in the motor cortical excitability, in contrast to the changes in healthy controls. Less severe patients
		11	54 ± 4	3 ± 1	11 ± 1	
Kishore et al. (64)	iTBS cTBS	17 (SR)	59 ± 3	4 ± 1	28 ± 3		ON and OFF	−	Three groups of patients with a spectrum of response to dopaminergic medication. Near normal LTP-like response to iTBS but decreased LTD-like response to cTBS in OFF medication state in all groups. l-DOPA normalizes LTD in the earlier stage of disease, which correlated with clinical improvement. In the patients with LID, l-DOPA reverses the direction of plasticity response with cTBS leading to paradoxical facilitation
		18 (FND)	56 ± 2	7 ± 1	32 ± 2			−	
		20 (FD)	56 ± 2	9 ± 1	44 ± 21			+	
**rTMS**									
Gilio et al. (65)	5 Hz rTMS	15	63 ± 2	–	23 ± 5		ON and OFF	−	Decreased facilitatory response in relaxed state in ON and OFF medication sessions, response during muscle contraction similar to controls
Lomarev et al. (66)	25 Hz	18	65 ± 10	–	22–39	2–4	ON and OFF	−	Increase in MEP amplitude after 8 sessions of rTMS
Buhmann et al. (67)	1 Hz (PMd)	10	58 ± 11	–	16 ± 6.9	2 ± 1	Single dose	No	1 Hz rTMS to PMd normalized the decreased short intracortical inhibition connection (5 ms) in drug naive PD. Similar results were obtained with first dose of l-DOPA

Dis dur, disease duration; DN, drug-naïve; FD, fluctuating dyskinetics; FND, fluctuating non-dyskinetics; H and Y, Hoehn and Yahr; LID, l-DOPA-induced dyskinesia; LTP, long-term potentiation; LTD, long-term depression, MEP, motor evoked potential; PD, Parkinson’s disease; PAS, paired associative stimulation (21.5 and 25 represents the latency in milliseconds between peripheral nerve stimulation and M1-transcranial magnetic stimulation), PMd, dorsal premotor cortex; rTMS, repetitive transcranial magnetic stimulation; SR, stable responders; TBS, theta burst stimulation; UPDRS, unified Parkinson’s disease rating scale.

## Effect of Dopaminergic Medications

In healthy humans, l-DOPA or dopamine agonists exert a powerful effect on M1 plasticity induced by rTMS (68), PAS and tDCS (69). The dopaminergic dose–plasticity response curve in healthy subjects has an inverted “U”-shape, in which low dopaminergic tone impairs plasticity, while moderate doses facilitate plasticity (69–71). Dopamine effects are different in two different plasticity-inducing protocols with diminution of plasticity following tDCS and stabilization or increase of PAS-induced plasticity. However, such non-linear relationship has not been investigated in PD. It has been observed that low (25 mg) and high (200 mg) dose of l-DOPA converts LTP-like plasticity of M1 (induced by PAS25) to LTD whereas moderate dose of l-DOPA (100 mg) potentiates the plasticity effects in healthy subjects (72). While low dose of dopamine activates presynaptic receptors and increases dopamine release, this reversal of plasticity at high doses (200 mg) was attributed to the high levels of D1 receptor stimulation which in turn inhibits NRs (72). Animal studies showed that dopamine directly regulates the induction of LTP and LTD in glutamatergic synapses in the striatum and the prefrontal cortex (73, 74). It is critical to activate the NRs situated on the membrane of MSN in the striatum to induce plastic changes in the corticostriatal synapse. At the molecular level, the interactions between dopamine and the NRs, the intracellular signal transduction of which requires a common integrator (α-CaMKII), control the striatal plasticity (28).

Although the exact cellular mechanism of the influence of dopamine on the M1 plasticity remains unknown, it is possible that dopamine modulates the dynamic circuitry of the cortical plasticity in the M1 through NRs. There are two possible dopaminergic pathways for modulating plasticity in the M1: an indirect nigrostriatal pathway via the BG-thalamocortical loop or a direct mesocorticolimbic pathway projected from the ventral tegmental area. In the nigrostriatal pathway, the motor cortical areas, including the M1, the supplementary motor area (SMA), the premotor area, and the cingulate motor areas project major glutamatergic fibers to the striatum, which belongs to a series of BG thalamocortical loops that project back to the motor cortex via the motor thalamic nuclei (75–78).

## Dyskinesias and Altered Plasticity

Following typically 5–10 years of l-DOPA therapy (79), PD patients may develop l-DOPA-induced dyskinesia (LID) characterized by purposeless, involuntary, repetitive movements which often reduce the quality of life (80). LID was associated with the loss of LTD expression at glutamatergic striatal spiny neuronal synapses and drugs that selectively targeting phosphodiesterases can ameliorate LID (81), possibly by restoring physiological synaptic plasticity in the striatum. LID in animal models are associated with an altered corticostriatal synaptic plasticity (29). In particular, in dyskinetic Parkinsonian animals the ability to reverse previously induced LTP is lost (27, 29).

## Depotentiation Studies in PD

Depotentiation represents a homoeostatic mechanism which allows the reversal of previously induced strengthening of synaptic efficacy in order to remove redundant synaptic information and, consequently, increase storage capability (17, 29). Picconi et al. (29) showed that dyskinetic rats lack this depotentiation ability compared to non-dyskinetic rats. Both group of rats showed potentiation following high-frequency stimulation but dyskinetic rats failed to show reversal to baseline excitability following subsequent low-frequency stimulation. Both dyskinetic and non-dyskinetic Parkinsonian rats showed normal LTP following chronic l-DOPA treatment. In PD patients, a study (82) using a depotentiation protocol that consisted of cTBS 150 (shorter protocol which has no direct effect by itself compared to cTBS300 which has LTD-like effects) showed that PD patients with LID failed to show depotentiation, which was present in healthy subjects and PD patients without LID. This loss of depotentiation could be attributed to an inability to erase unwanted motor information leading to aberrant abnormal motor pattern seen in dyskinesias. This lack of depotentiation may be due to changes occurring along the D1 dopamine receptor signaling pathway leading to abnormally high levels of Thr34-phosphorylated proteins (DARPP-32) and subsequent inhibition of protein phosphatase activity (17).

In animal models of LID, enhanced activation of the striatal glutamate receptors, particularly the NMDAR subtype, appears to be a major factor in the expression of dyskinetic movements (83). Variations in the organization of NR subunits (decreased NR2B and NR1A without change in NR2A) are closely associated with altered synaptic plasticity and are believed to be the cornerstone in the pathophysiology of LID in PD (41). In the normal physiological state, NRs localized to synaptosomal membranes are comprised of heterodimeric NR1/NR2A and NR1/NR2B receptors, and heterotrimeric NR1/NR2A/NR2B receptors (84). Following dopamine depletion in the Parkinsonian state, there is a selective reduction of NR1/NR2B heterodimeric receptors. This results in a relative enrichment of NRs containing NR2A subunits. Following repeated l-DOPA treatment causing LID, there is normalization in the level of NRs comprised of NR1 and NR2B subunits and an increase in NRs containing NR2A subunits. This increase in NR2A-containing NRs may bring about important changes to NR-mediated signaling in dyskinesia (85).

## M1 Plasticity Studies in PD

Most plasticity studies using TMS protocols showed impaired M1 plasticity in PD (Table 1). However, there are discrepancies which could be attributed to factors such as the age of the patient, disease duration, side of involvement (in asymmetric PD), dopaminergic medications, and the TMS protocol used to explore plasticity. We discuss these issues in the following sections.

### PAS protocols

When PAS was used to assess the M1 plasticity in PD patients, impairment of plasticity was observed in most studies (51, 52, 55–57). However, two studies showed exaggerated plasticity on the less affected side in drug-naïve patients (55) and in the OFF medication condition, which was normalized with dopaminergic medications (53). In the study of Kojovic et al. (55), this exaggerated plasticity observed on the less affected side was associated with less severe clinical involvement. Thus, this increased plasticity may represent compensatory changes on the less affected side in early PD. Since only drug-naïve patients were studied, it would be interesting to explore the short and long-term effects of dopaminergic medications on this plasticity. In the study of Bagnato et al. (53), exaggerated plasticity in PD with overflow to neighboring muscles (heterotopic plasticity) was observed in the OFF medication condition, which was normalized with dopaminergic medications (53). However, the study patients were heterogeneous in terms of disease duration, l-DOPA dosage and development of LID. Also, the authors investigated the right side irrespective of the clinical involvement. Exaggerated plasticity was also seen in tremulous patients with “scans without evidence of dopaminergic deficit (SWEDD)” (54), compared to deficient plasticity observed in PD patients with dopaminergic deficit. This study suggests that SWEDD patients may be closer to dystonia in terms of electrophysiological response to plasticity protocols than PD. Hence, plasticity response may differ in PD depending on subgroups and stages of PD.

Dopaminergic medications modulate the altered plasticity in PD (51, 52). This restoration correlated with decreased plasticity and disease severity as measured by Unified Parkinson’s Disease Rating Scale (UPDRS) scores (52). Furthermore, only PD patients without dyskinesias showed restoration of M1 plasticity by l-DOPA (51). However, the history of dopaminergic therapy *per se* may not have any effects on modulating the plasticity as shown by a study (56) that compared drug-naïve PD patients with those on l-DOPA and dopamine agonists in their plasticity response induced by PAS. They showed that M1 plasticity is equally deficient in both groups compared to healthy controls. However, the study was performed only in OFF medication condition without exploring the acute effects of dopaminergic medication (ON medication condition). Hence, the acute (ON) effects of dopaminergic medications may be required to restore the altered PAS-induced M1 plasticity in PD in the earlier stages of the disease.

In the later stage of PD, deficient plasticity in patients with LID was not restored by dopaminergic medications (51). A recent study (57) showed the deficient PAS-induced plasticity in PD patients with LID is restored by inhibitory cTBS to the cerebellum. The authors showed this restoration may be due to modulation of sensory input as only PAS but not iTBS induced M1 plasticity impairment was restored by cerebellar cTBS. In patients with LID treated with STN DBS, optimal stimulation in the on medication condition also restored PAS-induced M1 plasticity to normal level (86). Thus, M1 plasticity in PD changes with the phase of the disease with compensatory exaggeration on the unaffected side in early PD to deficient plasticity in later stages of PD, which may be restored with dopaminergic drugs and STN DBS. Furthermore, a study found involvement of cerebellar circuits in longer latency PAS (PAS25) and but not in shorter latency PAS21.5 [median nerve stimulation followed by TMS 21.5 ms later, Ref. (87)]. Since the neuronal circuits that mediate these two latencies of PAS are likely different, this may account some of the different results in previous studies. Further studies are required to elucidate the mechanisms and circuits involved in these two different latencies of PAS in PD.

### TBS protocols

Several studies have shown impaired plasticity in PD patients using TBS protocols. Kishore et al. (63) examined 10 drug-naïve PD patients with cTBS and iTBS. Both protocols failed to elicit changes in M1 excitability PD in contrast to the changes observed in controls. Interestingly, the first dose of l-DOPA failed to modulate the plasticity in these patients and there was no significant difference between the more and less affected sides. Deficient iTBS induced LTP-like plasticity in PD was observed in other studies (58, 60, 61). In one study (60) following facilitatory iTBS, neither the medication state (ON and OFF), disease severity nor the presence of LID had any effect on this lack of potentiation, suggesting that the reduced M1 plasticity in PD is unrelated to dopaminergic therapy, disease severity, and the developments of LID. Similarly, instead of potentiation, iTBS produced no change in excitability in eight patients ON dopaminergic drugs (61). In another study (58), inhibitory cTBS on M1 did not change in M1 excitability in PD patients. Another study (64) demonstrated altered plasticity in both early and late stages of PD and different effects of dopaminergic medications at different stages of the disease. In this study, patients were grouped into “stable responders,” “fluctuating non-dyskinetics,” and “fluctuating dyskinetics” based on their clinical response to dopaminergic medications. In OFF medication condition, LTP-like plasticity induced by iTBS was present, although at a lower level compared to healthy subjects in all patient groups. In contrast, LTD-like plasticity induced by cTBS was normal only in the stable response group, while it was reduced in the fluctuating non-dyskinetic group and was absent in the fluctuating dyskinetic group. In the ON medication conditions, l-DOPA in the stable responder group led to increased LTP induced by iTBS (to normal levels). On the other hand, in the fluctuating non-dyskinetic group, l-DOPA decreased LTP, and in the fluctuating dyskinetic group, no changes were observed following l-DOPA. There was reversal of the depression (toward LTP) induced by cTBS in all three groups following the medication. This reversal of plasticity negatively correlated with clinical effects of l-DOPA especially in patients with motor complications (fluctuating non-dyskinetics and fluctuating dyskinetics). Thus, l-DOPA modulation of cTBS induced M1 plasticity increases with disease progression and was associated with the development of LID.

On the other hand, another study (62) showed normal response to iTBS in PD in both on and off medication states compared to controls. Several factors may be responsible for these different results such as heterogeneous clinical features (different disease severity, patients with and without LID) and the highly variable response to TBS even in healthy subjects (88). Another study administered iTBS in PD patients and found increased cortical excitability after the first iTBS session, but no change in cortical excitability or motor symptoms after 2 weeks of treatment (59). Furthermore, studies in hemiparkinsonian rats (89) found restoration of LTD at corticostriatal synapses using iTBS to M1. Thus the results of M1 plasticity in PD using TBS protocols are variable.

### rTMS protocols

Several studies used rTMS to the M1 and other cortical regions to modulate their excitability and to alleviate PD symptoms. Only studies that assessed the M1 excitability changes will be discussed here. A study using high-frequency (5 Hz) rTMS on M1 (65) during muscle relaxation showed that PD patients failed to show MEP facilitation irrespective of the medication state (ON or OFF). These findings were different from control subjects who showed progressive increase in M1 excitability with trains of 5 Hz rTMS. However, when the study was performed during muscle contraction, there were no changes in cortical excitability in either the PD or control groups. Thus, this study showed decreased facilitatory response to LTP-like induction protocol in PD compared to controls in the relaxed state. This result is different from increased MEP amplitude induced by eight sessions of 25 Hz rTMS to bilateral M1 and dorsolateral prefrontal cortex (DLPFC) in PD patients with gait difficulties and LID (66). The clinical improvement with rTMS may be due to dopamine release, as dopamine level correlated with improvement in UPDRS scores (90).

rTMS was also used to explore the dorsal premotor (PMd)-M1 connections in PD. One study (67) investigated the PMd-M1 connection using 1 Hz rTMS to PMd in patients with early PD who were never treated with l-DOPA. This inhibitory protocol normalized the decreased intracortical inhibition of M1 tested with paired pulse protocol at 5 ms (with subthreshold conditioning and suprathreshold test stimuli to M1 with interstimulus interval of 5 ms). Similar results of increasing the cortical inhibition were obtained with the first dose of l-DOPA in medication naïve patients. Thus the authors concluded that 1 Hz rTMS to PMd modulates M1 intracortical circuits, probably by induction of dopamine release. In another study (91), 5 Hz rTMS of M1 alone did not change but when preconditioned with 5 Hz rTMS of PMd increased M1 excitability (measured by MEP amplitudes before and after the protocol) in the ON medication state. Since, such modulation occurred only with ON and not with OFF medication state, the authors concluded that dopamine restored the short-term plasticity of M1 by modulating PMd-M1 connectivity.

## Transcranial Direct Current Stimulation

Studies using tDCS have found that anodal M1 stimulation increased cortical excitability and cathodal stimulation decreased cortical excitability in PD (92). In addition to increasing the M1 excitability, anodal tDCS improved motor signs assessed with UPDRS, which showed a trend of correlation with excitability changes. Although healthy control group was not employed in this study, the polarity specific direction of change of excitability was similar to that reported in normal subjects (39). tDCS has been used to investigate the modulation of plasticity by dopaminergic medications and metaplasticity (69) as l-DOPA decreased anodal tDCS induced potentiation and increased cathodal tDCS induced depression of cortical excitability. On the other hand, l-DOPA had inverse U-shaped dose response curve for potentiation induced by PAS protocols [Ref. (72); more details in the section of “Effect of Dopaminergic Medications”]. These differential effects of l-DOPA on different plasticity protocols are further explored by Monte-Silva et al. (71) who examined modulation of plasticity by D2/D3 receptor agonist ropinirole in healthy volunteers. They found that high and low doses of ropinirole diminished plasticity induced by either PAS or tDCS whereas a medium dose potentiated the effect of plasticity induced by these protocols, producing parabolic dose-dependency. tDCS have also used to examine metaplasticity, as discussed in earlier sections. A study (93) showed the metaplasticity effects of both cathodal and anodal tDCS and 1 Hz rTMS on various kinematic measures in PD. Cathodal tDCS followed by 1 Hz rTMS improved finger pointing movements, whereas 1 Hz rTMS preconditioned by anodal tDCS showed no such benefits. However, cortical excitability was not examined in this study. Thus, tDCS is a simple non-invasive technique to study metaplasticity and the effects of dopaminergic drugs on cortical plasticity in PD.

## Future Studies in PD-M1 Plasticity Study

Because of the asymmetric involvement of clinical features and plasticity in the M1 of PD patients (55), it would be ideal to conduct a prospective study in a population at high risk of developing PD (e.g., LRRK-2 mutation carriers). Baseline plasticity measures may be obtained on both the hemispheres in the preclinical stage and the participants followed throughout the course of development of PD. This would define the utility of these M1 plasticity measures as prognostic or diagnostic tests and clarify the pathophysiology of M1 plasticity in PD. Furthermore, with a long-term prospective design, l-DOPA response and subsequent development of LID could be investigated in this cohort based on the pharmacogenetic approach to link genetics and subsequent development of aberrant plasticity in BG (94).

## Role of Deep Brain Stimulation in Modulating M1 Plasticity

Deep brain stimulation of the BG structures such as STN and globus pallidus internus (GPi) represents a breakthrough in the management of late PD with motor complications (10). Although the exact mechanisms of actions DBS is not known, in addition to providing clinical benefits, DBS have improved our understanding of the BG and their connections with M1. By pairing STN-DBS and M1-TMS, it was found that M1 excitability is increased at two specific latencies of about 3 and 22 ms after STN DBS (95). The short latency facilitation (∼3 ms) is likely due to antidromic activation of the cortical-STN pathway as demonstrated by STN-DBS in anesthetized rats (96) where as the longer latency (∼23 ms) may be due to orthodromic conduction in the indirect pathway. In another study (97), it was shown that repeated pairing of STN-DBS and M1-TMS at these two specific latencies could induce M1 plasticity. Therefore, modulation of M1 plasticity could be one of the mechanisms of action of DBS. In addition, STN DBS together with dopaminergic medications restored PAS plasticity in advanced, dyskinetic PD patients (86).

The effects of l-DOPA on plasticity in the BG have been observed in PD patients undergoing DBS implantation (98). The authors used high-frequency stimulation to induce plasticity and recording the field evoked potentials in the substantia nigra pars reticulata using microelectrode. They found that little plasticity was induced in the OFF condition, which was increased by l-DOPA. Since the l-DOPA dose was very low (100 mg) in this study to avoid dyskinesias during the surgical procedure, the modulation of BG plasticity by dopaminergic treatment was only partially addressed. In addition, an optogenetic study showed that modifying the activity of STN neurons was less effective than direct cortical stimulation in reversing the movement deficits following 6-OHDA lesions in mice (99). Thus modifying M1 plasticity might offer therapeutic benefits in PD, and maybe one of the mechanisms of action of DBS.

## Therapeutic Strategies That Modulate M1 Plasticity by Non-Invasive Brain Stimulation

In early stages of PD, there is decreased activity in the medial motor areas such as the SMA whereas hyperactivity was found in more lateral regions such as the M1 in more advanced stages of the disease (100, 101). Non-invasive brain stimulation techniques that alter the plasticity of these cortical-subcortical networks have been tested as treatment of PD. In early PD, rTMS in single (102, 103) and multiple session (104) designs as well as anodal tDCS (92, 105, 106) showed variable improvement in PD symptoms. Further, in more advanced PD, low-frequency rTMS to SMA (107), cerebellum (108), and M1 (109, 110) transiently improve LID. Meta-analyses (111, 112) of rTMS studies in PD found significant improvement in PD motor symptoms with high-frequency rTMS to M1. Thus, modulation of M1 excitability in PD has therapeutic potential. This may be further explored with pairing M1 stimulation with stimulation of BG structures such as STN and GPi DBS, other cortical structures (SMA, DLPFC, and other cortical areas involved in PD) to further increase the clinical benefits. Furthermore, studies that investigate other neurotransmitter pathways such as cholinergic, adrenergic, and serotonergic systems with pharmacological agents and neuroimaging techniques will further our understanding of the pathophysiology of BG and M1 synaptic plasticity in PD. This will help to develop new modes of investigations to further understand the disease and identify therapeutic targets for effective management of PD.

## Conflict of Interest Statement

The authors declare that the research was conducted in the absence of any commercial or financial relationships that could be construed as a potential conflict of interest.
